# Supplementary material for: Content-rich biological network constructed by mining PubMed abstracts
Source: BMC Bioinformatics. 2004 Oct 8;5:147. doi: 10.1186/1471-2105-5-147 (PMC528731; doi:10.1186/1471-2105-5-147)
Supplement: Additional File 2 — The original results of the above study (non-essential files are deleted to keep the file size under the limit set by BMC bioinformatics). [file 1471-2105-5-147-S2.bz2 › chilibotAdditionalFile2/dip30/436ID7545673E10381/html/CCR1_CCL7.html]

 


 **CCR1** and **CCL7** 
  
Found 54 abstracts in PubMed, retrieved 30.  
 

 What does Google say? 
 PDF only 
| .edu only 

---

**Interactive relationship** (e.g. stimulation, inhibition, etc)

**Inhibitory relationship**- In a parallel manner, binding of labeled MIP 1alpha was first shown to be attenuated by unlabeled RANTES, both of which recognize  **CCR1**  and CCR5, and then separately antagonized by MCP 3  [ **CCL7** ]  and MIP 1beta, which bind to  **CCR1**  and CCR5, respectively.  Ref: 10559781 Glia, 1999
**Neutral relationship**- Labeled MIP 1alpha binding was also antagonized by unlabeled MCP 3  [ **CCL7** ] , which is also recognized by  **CCR1** , and MIP 1beta, which is a ligand for CCR5.  Ref: 11032879 J Neurochem, 2000
- Monocyte chemotactic protein 3  [ **CCL7** ]  MCP 3  [ **CCL7** ]  is a C C chemokine that interacts with the  **CCR1** , CCR2, and CCR3 receptors and has a spectrum of action encompassing T cells, NK cells, eosinophils, and dendritic cells DC, in addition to mononuclear phagocytes.  Ref: 9647242 J Immunol, 1998
- Expression of L CCR  [ **CCR1** ]  in HEK 293 cells reveals functional responses to CCL2, CCL5,  **CCL7** , and CCL8.  Ref: 12885941 J Leukoc Biol, 2003

**Non-interactive relationship** (e.g. studied together, co-existance, homology, etc.)

- We used radioligand binding and 35S GTPgammaS exchange assays in membranes from a cell line transfected to express  **CCR1**  Ba F3 hCCR1 to characterize a panel of chemokines HCC 1, MIP 1alpha, MIP 1beta, MIP 1delta, MPIF 1, MCP 2, MCP 3  [ **CCL7** ] , and RANTES as  **CCR1**  ligands.  Ref: 12381680 Br J Pharmacol, 2002
- Competition binding studies revealed that BX 471 was able to displace the  **CCR1**  ligands macrophage inflammatory protein 1alpha MIP 1alpha, RANTES, and monocyte chemotactic protein 3  [ **CCL7** ]  MCP 3  [ **CCL7** ]  with high affinity K i ranged from 1 nm to 5.5 nm.  Ref: 10748002 J Biol Chem, 2000
- GM CSF induced up regulation of  **CCR1**  showed functional consequences because GM CSF treated PMN, but not control cells, responded to the CC chemokines macrophage inflammatory protein 1alpha, monocyte chemoattractant protein 3  [ **CCL7** ] , and RANTES in assays of chemotactic migration and intracellular calcium mobilization.  Ref: 11145699 J Immunol, 2001
- At 26 weeks postirradiation, messages encoding the chemokines BLC now known as Scyb13, C10 now known as Scya6  [ **CCL7** ] , IP 10 now known as Scyb10, MCP 1 now known as Scya2, MCP 3  [ **CCL7** ]  now known as Scya7  [ **CCL7** ] , MIP 1gamma now known as Scya9, and RANTES now known as Scya5 and the chemokine receptors  **Ccr1** , Ccr2, Ccr5 and Ccr6 were elevated in fibrosis sensitive C57BL 6 mice.  Ref: 11839087 Radiat Res, 2002
